# Systematic Review on White Spot Lesions Treatments

**DOI:** 10.1055/s-0041-1731931

**Published:** 2021-08-27

**Authors:** Francesco Puleio, Luca Fiorillo, Francesca Gorassini, Alfredo Iandolo, Aida Meto, Cesare D’Amico, Gabriele Cervino, Mirta Pinizzotto, Giancarlo Bruno, Marco Portelli, Alessandra Amato, Roberto Lo Giudice

**Affiliations:** 1 Department of Biomedical and Dental Sciences and Morphofunctional Imaging, Messina University, Messina, Italy; 2 Multidisciplinary Department of Medical-Surgical and Odontostomatological Specialties, University of Campania “Luigi Vanvitelli,” Naples, Italy; 3 Department of Implantology, Faculty of Dentistry, University of Aldent, Tirana, Albania; 4 Department of Medicine and Surgery, University of Salerno, Salerno, Italy; 5 Department of Dental Therapy, Faculty of Dental Medicine, University of Medicine, Tirana, Albania; 6 Department of Clinical and Experimental Medicine, Messina University, Messina, Italy

**Keywords:** white spot lesion, resin infiltration, enamel demineralization, aesthetics, enamel remineralization, microabrasion

## Abstract

The difference in refractive index between the healthy enamel and the demineralized area generates a lesion with a milky white opaque appearance, clearly distinguishable from the surrounding healthy enamel. The aim of this systematic review was to evaluate if the infiltration technique is the most efficient treatment to resolve a white spot lesion when compared with remineralization and microabrasion techniques. The Population/Intervention/Comparison/Outcome question investigated: “in enamel WS lesion, the infiltration treatment compared to remineralization or microabrasion treatments is more or less effective in the camouflage effect?.” The research was performed on electronic databases, including Ovid MEDLINE, PubMed, and web of science. The search was conducted up to April 1, 2020. The scientific search engines produced 324 results. Only 14 were screened after screening. Based on the articles analyzed in this systematic review, the resin infiltration technique seems to be the most effective and predictable treatment for the aesthetic resolution of WSLs.

## Introduction

The enamel translucency is a characteristic related to the composition of the inter-crystalline space and could be quantitatively defined by the enamel refractive index (ERI). 1

Therefore, any clinical situation that leads to an alteration of the enamel organization determines a variation of the ERI (1.62). 1

The difference in refractive index between the healthy enamel and the demineralized area generates a lesion with a milky white opaque appearance, clearly distinguishable from the surrounding healthy enamel. 2 3

The white spot lesions (WSLs) pathogenesis may be various. The main cause is related to an overtime plaque accumulation; moreover, many other factors as diet and levels of calcium, phosphate, bicarbonate, fluoride in saliva as well as genetic factors are reported. 4

The WSLs are a frequent finding in patients with fixed orthodontic treatments (46%) due to plaque retention caused by presence of brackets and bands. 5

Several treatments have been described in the literature to prevent the possible WSL progression and the cavitation and dyschromia appearance related.

Microabrasion could improve teeth aesthetic eliminating the outer defective enamel layer. This invasive technique uses 6.6% hydrochloric acid and 20- to 160-μm sized silicon carbide microparticles to remove superficial parts of the lesion. 6 7

Remineralizing agents containing 5% fluorine or casein phosphopeptide (CPP-ACP) could be used as noninvasive treatments in the early stages of WSL. 8 9 10 11 This minimally invasive approach does not solve the aesthetic problem in advanced lesions due to the limited infiltrating capacity of the agent that act in the enamel external part and therefore could result in an untreated discolored area. 12 13

The resin infiltration technique (RIT) consists in etching with a 15% hydrochloric acid that increases enamel porosity followed by the infiltration of a highly viscous and highly penetrating resin in the thickness of WSLs.

The resin stops the progression of the WSLs and creates a barrier against further cariogenic attacks. 14 The resin refractive index is similar to the ERI one and masks the opaque white appearance typical of WSLs.

The resolution of these lesions exploits the camouflage effect resulted from different techniques to mask the dichromatism and to obtain an additional aesthetical clinical success.

The aim of this systematic review was to evaluate if the infiltration technique is the most efficient treatment to resolve a WSL when compared with remineralization and microabrasion techniques.

## Methods

### Protocol and Registration

This systematic review was conducted according to the guidelines of the Preferred Reporting Items of Systematic Reviews and Meta-Analyses (PRISMA) statement. 15 Before starting the review, a detailed protocol of the methodology was developed. The review was registered in the CRD York website PROSPERO. The protocol number is CRD42020164187.

### Search Strategy

The research was performed on electronic databases, including Ovid MEDLINE, PubMed, and web of science. The search was conducted up to April 1, 2020.

The following terms and their combination were searched: “White Spot,” “Resin Infiltration,” “Remineralization,” and “Microabrasion.” The choice of keywords was intended to collect and to record as much relevant data. The research was conducted by using a search formula as follows:

(((((white spot) AND resin infiltration) OR white spot) AND remineralization) OR white spot) AND microabrasion.

The following focus question was developed according to the population, intervention, comparison, and outcome (PICO) study design:

“In enamel WS lesion, the infiltration treatment compared to remineralization or microabrasion treatments is more or less effective in the camouflage effect?.”

The review included randomized clinical trial and in vitro studies that compared the results of the RIT to remineralization and microabrasion techniques for the WSL treatment.

Only studies published between January 2013 and April 2020 were considered.

### Eligibility Criteria

The full texts of all possibly relevant studies were selected considering the following inclusion criteria:

- Study that compared the results of WSLs treatment using the infiltration technique to remineralization and microabrasion techniques. Human trial (randomized controlled trial and clinical trial) and in vitro study.
  The exclusion criteria applied to the following studies:
- Studies involving patients with dental diseases (e.g., hypocalcification, hypoplasia, fluorosis, and hypoplasic molar-incisive syndrome)
- Studies that use infiltration technique only
- Studies that not compare infiltration technique to remineralization and microabrasion techniques
- Studies that compare RIT with other restorative materials (flow composite and adhesive system)
- Case report, case series, review, and meta-analysis
- Papers without full text available

### Risk of Bias Assessment

The evaluation of in-vitro studies was based on a methodological index that use a checklist for in-vitro studies on dental materials (CONSORT). The checklist items focus on reporting how the study was designed, analyzed, and interpreted by using 14 domains. 16 Randomized clinical trials were assessed according to the modified Cochrane Collaboration. 17 Bias is assessed as a judgment (high, low, or unclear) for individual elements from five domains (selection, performance, attrition, reporting, and other).

## Results

### Study Selection

**Fig. 1:**
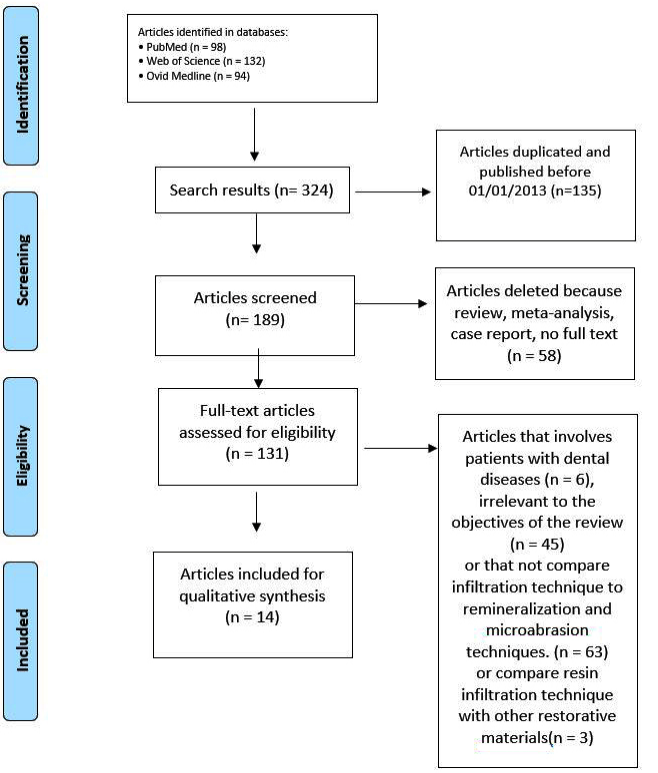
Preferred Reporting Items for Systematic Reviews and Meta-Analyses flow chart.

The scientific search engines produced 324 results. The duplicates were eliminated, obtaining a total number of 189 studies. Furthermore, 58 articles were deleted because review, meta-analysis, case report, or not full text by reading the abstract. After the first screening, 131 studies were subjected to a full-text examination. Of 131 articles, 6 were discarded because involved patients with dental diseases (e.g., hypocalcification, hypoplasia, fluorosis, and hypoplastic molar-incisor syndrome), 45 because irrelevant to the review’s objectives, 63 because does not compared RIT with any other techniques, 3 because compared RIT with other restorative materials. Fourteen studies were included in this review 18 19 20 21 22 23 24 25 26 27 28 29 30 31 ( Fig. 1 ). The selected studies are listed in Table 1 .

**Table 1:** Data extraction from selected studies

Study (Year)	Object of research	Intervention	Evaluation methods	Result
Behrouzi et al (2020) 18	45 maxillary central incisors	RA, RI	Vickers hardness test	Color: RA: ( *p* < 0.01) RI: ( *p* > 0.05)
Torres et al (2019) 19	80 flat enamel disks from bovine incisors	Co, RA, RI	Spectrophotometer	Color: RA: ( *p* > 0.05) RI: ( *p* < 0.01)
Yadav et al (2019) 20	72 extracted premolars	Co, RA, RI	Spectrophotometer and laser fluorescence	Fluorescence: RA, RI: ( *p* > 0.05) Color: RA, RI ( *p* > 0.05) RI vs. RA: ( *p* < 0.01)
Arora et al (2019) 21	120 premolars	Co, RA, RI	Profilometer Vickers hardness tester	Surface roughness: RA, RI: ( *p* < 0.01) RI vs. RA: ( *p* < 0.01) Depth of penetration: RA, RI: ( *p* < 0.01) RI vs. RA: ( *p* < 0.01) Microhardness: RI: ( *p* < 0.01) RA: ( *p* > 0.05) RI vs. RA: ( *p* < 0.01)
Silva et al (2018) 22	Bovine incisors	Co, RI, MA	Spectrophotometer	Color: RA, RI: ( *p* > 0.05)
Krishna et al (2018) 23	90 maxillary permanent central incisors	RA, RI	Spectrophotometer	Color: RI: ( *p* < 0.001) RA: ( *p* > 0.05) RI vs. RA: ( *p* < 0.001)
Wierichs et al (2017) 24	300 enamel blocks from bovine incisors	Co, RA, RI	Transversal microradiographic images, digital photographs, spectrophotometer	Depth of penetration: RI: ( *p* < 0.01) RA: ( *p* > 0.05) Color: RI: ( *p* < 0.05) RI: ( *p* < 0.01) RI vs. RA: ( *p* < 0.05)
Yuan et al (2014) 25	52 premolars and molars	Co, RA, RI	Spectrophotometer, fluorescence	Color: RI: ( *p* < 0.01) RA: ( *p* > 0.05) Co: ( *p* > 0.05) Fluorescence: RI: ( *p* < 0.01) RA: ( *p* > 0.05) Co: ( *p* > 0.05)
Yetkiner et al (2014) 26	96 bovine teeth	Co, RA, RI, MA	Spectrophotometer	Color: Co: ( *p* > 0.0033) RA: ( *p* > 0.0033) RI: ( *p* < 0.0033) MA: ( *p* < 0.0033)
Kannan et al (2019) 27	240 WSLs in 193 postorthodontic teeth from 12 patients	RI, RA	Spectrophotometer	Color: RA: ( *p* < 0.01) RI: ( *p* < 0.01) RA vs. RI: ( *p* < 0.01) Fluorescence: RA: ( *p* < 0.01) RI: ( *p* < 0.01)
Gu et al (2019) 28	108 WSLs from 16 postorthodontic patients with debonding more than 3 mo previously	RI, MA	Spectrophotometer	Color: RA: ( *p* < 0.001) RI: ( *p* < 0.001) RI vs. RA: ( *p* < 0.05)
Gözetici et al (2019) 29	113 WSLs from 319 patients	Co, RA, RI	LAA-ICDAS and laser fluorescence	Color: Co: ( *p* > 0.05) RA: ( *p* < 0.05) RI: ( *p* < 0.001) RI vs. RA: ( *p* < 0.05)
Giray et al (2018) 30	81 anterior WSLs from 23 patients	RI, RA	Laser fluorescence	Color: RA: ( *p* < 0.05) RI: ( *p* < 0.05) RI vs. RA: ( *p* < 0.05)
Ciftci et al (2018) 31	WSLs in 132 teeth		Laser fluorescence and ICDAS II scores	Color: RA: ( *p* < 0.001) RI: ( *p* < 0.001) ICDAS II scores RA: ( *p* < 0.05) RI: ( *p* < 0.001)
Abbreviations: Co, control; LAA-ICDAS, Lesion Activity Assessment-International Caries Detection and Assessment System; MA, microabrasion; RA, remineralizing agent; RI, resin infiltration; WSL, white spot lesion.				

### Risk of Bias

Tables 2 and 3 present the risk of bias of the in vitro studies and randomized clinical trials (RCT).

**Table 2:** Summary of the risk of bias for in-vitro studies according to Consolidated Standards of Reporting Trials

Item	Behrouzi et al (2020) 18	Torreset al (2019) 19	Yadav et al (2019) 20	Arora et al (2019) 21	Silva et al (2018) 22	Krishna et al (2018) 23	Wierich et al (2017) 24	Yuan et al (2014) 25	Yetkiner et al (2014) 26
1 Abstract	Yes	Yes	Yes	Yes	Yes	Yes	Yes	Yes	Yes
2a Background and objectives	Yes	Yes	Yes	Yes	Yes	Yes	Yes	Yes	Yes
2b Background and objectives	Yes	Yes	Yes	Yes	Yes	Yes	Yes	Yes	Yes
3 Intervention	Yes	Yes	Yes	Yes	Yes	Yes	Yes	Yes	Yes
4 Outcomes	Yes	Yes	No	No	Yes	No	Yes	Yes	Yes
5 Sample size	No	No	Yes	No	Yes	No	Yes	No	Yes
6 Randomization: sequence generation	No	No	No	No	No	No	No	No	No
7 Allocation concealment mechanism	No	No	No	No	No	No	No	No	No
8 Implementation	No	No	No	No	No	No	No	No	No
9 Blinding	No	No	No	No	No	No	No	No	No
10 Statistical methods	Yes	Yes	Yes	No	Yes	Yes	Yes	Yes	Yes
11 Results: outcomes and estimation	Yes	Yes	No	Yes	Yes	Yes	Yes	Yes	Yes
12 Discussion: limitations	Yes	No	Yes	Yes	Yes	Yes	Yes	Yes	Yes
13 Other information: funding	No	No	No	Yes	Yes	Yes	Yes	No	No
14 Protocol	Yes	No	Yes	Yes	Yes	Yes	Yes	Yes	Yes

**Table 3:** Summary of the risk of bias for randomized controlled trial studies according to the Cochrane Collaboration tool for assessing risk of bias

Item	Kannan et al (2019) 27	Xi et al (2019) 28	Gözetici et al (2019) 29	Giray et al (2018) 30	Ciftci et al (2018) 31
Random sequence generation	Low	Low	Low	Low	Unclear
Allocation concealment	Low	Low	Low	Unclear	Unclear
Selective reporting	Low	Low	Low	Low	Low
Blinding (participants and personnel)	High	high	high	High	High
Blinding (outcome assessment)	High	High	High	High	High
Incomplete outcome data	Low	Low	Low	Low	Low

## Discussion

The studies examined in this review mainly consist in-vitro studies. Five randomized clinical studies were also found. The risk of bias for these studies is presented in Tables 2 and 3 . Ten studies were considered as having a high risk of bias, mainly due to lack of random sequence generation and blinded investigator, potentially introducing selection bias. 18 19 20 21 22 23 24 25 26 31 Only four studies were assessed as having a low risk of bias. 27 28 29 30

To evaluate how different treatments modify the clinical outcome for the resolution of WSLs, different combinations of evaluation methods and clinical parameters were compared in the articles included in this review.

The authors of the included studies used different evaluation methods, such as spectrophotometry, digital camera combined with software analysis, and laser fluorescence, to evaluate the effect of various treatments on WSLs.

The researches included, investigated various parameters alone or in combination as clinical outcome, such as color change, superficial roughness alteration, microhardness alterations, ability to stop the WSL progression, and penetration depth of the treatment. These authors used different detection methods such as optical profilometer, confocal laser, and transverse microradiography.

All the in vivo studies evaluated the aesthetic resolution of the lesion, showing a significant regression of WSLs using RIT (ICON), remineralizing agents, and microabrasion. The lesions treated with RIT had a statistically significant improvement in camouflage effect, compared with those treated with fluoride varnish. 30 31 Although using a varnish with a very high concentration of fluoride (22,600 ppm) the lesions treated with RIT still show a significantly greater color change. 29 Microabrasion improves the aesthetic appearance of WSLs, but with a significantly less refractive index reduction than the infiltration technique; moreover, the results obtained with the resin infiltration also remain stable after 12 months, while the lesions treated with microabrasion tend to recur. 28 Turska-Szybka et al showed that it is possible to improve the results obtained using a fluoride varnish if a RIT treatment is also added. 32

In another study, RIT demonstrated a significantly better outcome than a resin-modified glass ionomer remineralizing agent (fluoride varnish), but after 3 and/or 6 months, the WSLs returned to be visible; however, lesion treated with fluoride varnish shows a superior long-term stability. 27

Nine in vitro studies were included in our systematic review. 18 19 20 21 22 23 24 25 26 Attia et al. used bovine dental elements because these substrates have a similar behavior regarding staining effects. 33 The in vitro studies analyzed does not concord among them when comparing their findings regarding the aesthetic results; moreover, one reported the failure of both RIT and remineralizing agents treatment for WSLs treatment.

When evaluating the aesthetic results, some of these studies demonstrate a better outcome obtained using RIT instead of remineralizing agents such as fluorinated solutions (with a more or less high percentage of fluorine), CPP-ACP, self-assembling peptide P11–4, and microabrasion. 19 23 24 25 26

Silva et al conclude that both RIT infiltration and microabrasion were not able to restore the tooth color. 22

However, it should be noted that the etching technique used in this research (15% HCl for 2') was probably insufficient. The company suggests to repeat the application up to a maximum of three times lesions is still evident after the first etching agent application.

Some authors reported that the number of etching applications can be correlated to WSLs characteristics. Wide, deep, smooth, and shiny lesions need more etching steps, and they might remain visible after resin infiltration. 34 35

### Depth of Penetration

Some studies included in our review have analyzed the aesthetic results linked to penetration ability of the resin and remineralizing agents.

Arora et al reported that fluorinated varnish cannot penetrate enamel as deeply as RIT. 21 The same result is confirmed by Rosianu et al; they show how 5% fluoride gel topical application does not remineralize the deep layers of the lesion. These authors state that the RITs are more efficient in deep layer infiltration of WSLs. 36

The 15% hydrochloric acid required in the RIT, allows an enamel etching deeper than the orthophosphoric acid used in other remineralizing techniques. 37 38

**Fig. 2:**
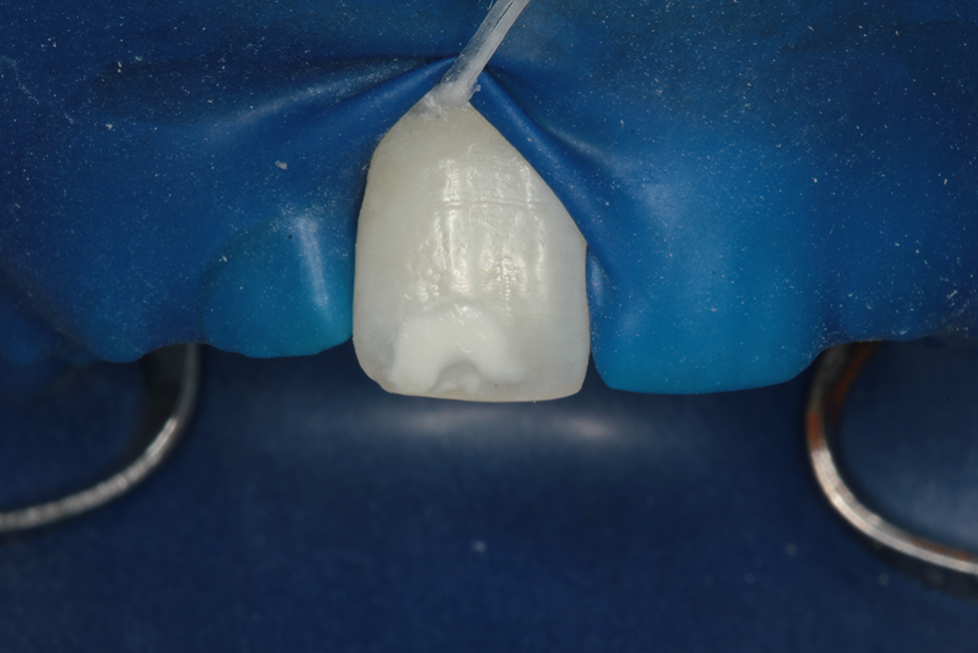
Dental isolation phase.

**Fig. 3:**
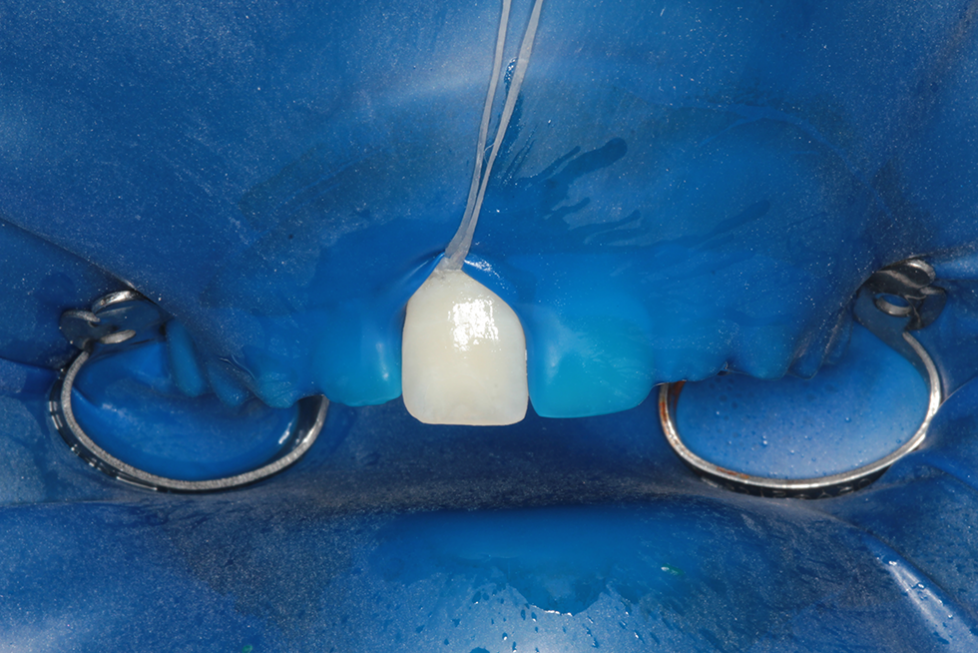
Result after resin infiltration.

According to Kane et al, the etching penetration allows a better infiltration of the resin in the treated enamel. The absence of gap inhibits the bacterial proliferation and WSLs progression 39 ( Figs. 2 and 3 ).

### Surface Microhardness

Two studies analyzed enamel microhardness variations subsequent to remineralizing or infiltrating treatment. 18 21

Behrouzi et al show how topic application of two fluoride gel (900 and 1450 ppm fluoride concentration) significatively increase the enamel microhardness; this effect was not reported by using RIT. 18

However, Arora et al showed a significant hardness increase of enamel infiltrated with resin compared with the one treated with remineralizing sealants. Arora et al concluded that the resin fills the lesion and improves the mechanical strength. 21

### Enamel Roughness

Arora et al showed that resin infiltration leaves the glazed surface smoother, while any modification is observed by using fluoride varnish. 21

Arnold et al in accordance with this study confirms that the infiltrated tooth surface is smoother, making more difficult plaque adhesion. 40

### Water Absorption

Some authors have investigated the ability of treated enamel to avoid the pigmentation.

In the studies included in this review, RIT was more susceptible to pigmentation than any other technique evaluated. 22 25

The resin used for infiltration is mainly composed by TEGDMA. It possesses a higher capacity of water absorption than BisGMA and UDMA. 41 42 This property has been correlated to a possible late pigmentation due to water carrier effect for pigments. 43 44

To prevent color alteration overtime, some authors suggest to repeat the polishing phase of the treated surface over time. An alternative is the walking bleach technique with carbamide peroxide. 45 46

The results of in vivo prospective studies, in contrast, do not show WSLs pigmentations when treated with RIT. But these studies limitation is the short follow-up (1 year). 34 47 48 49 50 51 52 53 54 55 . This process changes the refractive index in the light of the treated area (healthy enamel, normal, and hydrated) by saliva has a refractive index of 1.62, while the demineralized one of the white spots is between 1.00 and 1.33. By treating the defect with resinous infiltration, the enamel acquires an index equal to 1.52: a figure very close to that of healthy enamel, with a slight difference not perceptible to the human eye.

In other words, this treatment allows you to modify the interaction of light with the enamel and therefore the visual perception by the external observer.

It should be noted, however, that not all white enamel defects can be successfully applied: the deeper the white spot goes into the thickness of the enamel, the more “resistant” it will be to infiltration procedures. In the case of very deep and pigmented lesions—that is, which also have dark areas—this type of treatment may be of little or no effect.

Treatment alternatives can be identified in remineralization by using creams based on calcium and phosphate in casein matrix or in microabrasion of the enamel, even if the latter appears in some cases a risky procedure since it acts on the entire surface of the enamel and it can expose the entire tooth surface to a fall in value or brightness (generally, microabrasion is indicated for translucent enamel where the fall in value is contained). 56 57 58 59 60 61 62

Therefore, prospective studies with longer follow-up are needed to investigate the long-term stability of this treatment.

### Limitations

The first limitation of this study is linked to the different methods used to evaluate the color change. These differences produce noncomparable results in a meta-analysis. Another limitation of in vitro studies considered is the high risk of bias due to the lack of blinded investigator and random sequence generation methodology. No RCT with long follow-up are present to date.

## Conclusion

Based on the articles analyzed in this systematic review, the RIT seems to be the most effective and predictable treatment for the aesthetic resolution of WSLs. There is no strong evidence supporting microabrasion or remineralization technique. More RCT with a longer follow-up period are necessary to clarify the most effective approach for WSLs resolution.
